# Developing Advanced Quantum Materials is Key to Promoting Science and Technology

**DOI:** 10.1002/advs.202407326

**Published:** 2024-07-17

**Authors:** Yi Du, Shulei Chou, Run‐Wei Li

**Affiliations:** ^1^ School of Physics Beihang University Beijing 100191 China; ^2^ College of Chemistry and Materials Engineering Wenzhou University Wenzhou 325035 China; ^3^ Wenzhou Key Laboratory of Sodium‐Ion Batteries Wenzhou University Technology Innovation Institute for Carbon Neutralization Wenzhou 325035 China; ^4^ CAS Key Laboratory of Magnetic Materials and Devices Ningbo Institute of Materials Technology and Engineering Chinese Academy of Sciences Ningbo 315201 China

The exploration and development of advanced quantum materials represent a transformative leap in the realm of materials science. As we have delved deeper into the nanoscale and quantum domains, our understanding and manipulation of materials have reached unprecedented levels of precision and sophistication. This burgeoning field stands at the confluence of various disciplines, drawing expertise ranging from physics, chemistry, and engineering to propose materials with unique quantum mechanical properties that manifest at the atomic scale. Scientists are at the forefront of this revolution, meticulously engineering new classes of materials such as 2D materials, topological insulators, and quantum dots. These materials are not mere scientific curiosities; they hold the potential to revolutionize our technological landscape. For instance, 2D materials such as graphene boast exceptional strength and electrical conductivity, making them ideal for next‐generation electronic devices. Topological insulators, with their ability to conduct electricity on their surface while remaining insulating within, could pave the way for low‐power, high‐efficiency electronics. Quantum dots, with their size‐tunable electronic properties, are finding applications in quantum computing and as fluorescent markers in biomedical imaging. The implications of these materials extend far beyond the laboratory. Their integration into electronic systems, energy storage solutions, sensors, and quantum computing platforms is driving critical advances across multiple sectors. In quantum computing, the delicate manipulation of quantum states in materials is the key to developing powerful, scalable quantum processors that could solve complex problems beyond the reach of classical computers. In the realm of energy, advanced materials are instrumental in creating more efficient photovoltaic cells for solar panels, leading to cleaner and more sustainable energy sources. Moreover, the field of nanomaterials is witnessing the synthesis of novel substances aimed at energy conversion, such as photocatalysts that can harness sunlight to split water into hydrogen fuel. The development of eco‐friendly biodegradable polymers offers a promising solution to the plastic waste crisis, while biomimetic materials inspired by natural processes and structures are opening new avenues in medical and environmental applications. Researchers are not only harnessing the unique properties of these materials but are also addressing global challenges through sustainable innovation. The integration of advanced materials has already begun to reshape whole industries, leading to breakthroughs that enhance the capabilities of electronics, improve energy storage technologies, advance healthcare treatments, and contribute to environmental conservation efforts.

The field of advanced quantum materials holds immense promise for revolutionizing various technological domains, from energy to computing. These materials, characterized by their unique quantum mechanical properties, offer unprecedented opportunities for the development of new devices and systems with enhanced capabilities. The journey from laboratory discovery to practical application, however, is fraught with challenges. The synthesis of advanced quantum materials often involves complex procedures that require precise control over the material's composition and structure. The intricate crystal structures of these materials necessitate specialized fabrication techniques. For instance, the growth of single crystals, essential for understanding intrinsic properties, often requires methods such as molecular beam epitaxy or chemical vapor deposition, which are not only costly but also time‐consuming and technically demanding. Characterization techniques, too, are equally sophisticated. Equipment such as scanning tunneling microscopy (STM) or angle‐resolved photoemission spectroscopy (ARPES) are indispensable for probing the electronic structure and surface states of these materials. These methods usually require ultra‐high vacuum conditions, however, and are limited to surface analyses, leaving their bulk properties sometimes less understood. To truly harness the potential of advanced quantum materials, researchers must delve into the fundamental physics that governs their behavior. This includes a deep understanding of phenomena such as superconductivity and the exploration of topological insulators. Moreover, novel quantum effects, such as the quantum spin Hall effect, demand a thorough investigation to be effectively utilized in applications like spintronics. Researchers are tasked with finding ways to synthesize these materials in large quantities while maintaining their unique quantum properties. This often means rethinking traditional manufacturing processes and developing new ones that are both cost‐effective and efficient. Another vital aspect is the standardization of characterization and testing protocols.

This issue focuses on the synthesis of advanced quantum materials, highlighting the unique properties of these materials arising from the crystal structures that enable them to perform important functions in multiple fields, including optics, electronics, biology, and medicine. Studying these advanced materials requires interdisciplinary knowledge, including a combination of physics, chemistry, materials science, and engineering. We hope that researchers in various fields can focus on research on advanced quantum materials to promote progress in quantum materials science. Collaboration is key to overcoming the above challenges. By fostering partnerships between physicists, chemists, materials scientists, and engineers, as well as industry practitioners, this field can benefit from a multidisciplinary approach.

